# Supplementary material for: Risk Assessment of Postoperative Pneumonia in Cancer Patients Using a Common Data Model
Source: Cancers (Basel). 2022 Dec 4;14(23):5988. doi: 10.3390/cancers14235988 (PMC9740852; doi:10.3390/cancers14235988)
Supplement: Supplementary file 1 [file cancers-14-05988-s001.zip › cancers-2027743-supplementary.pdf]

Table S1. Risk factors for postoperative pneumonia in lung cancer and gastric cancer.

Variables	Lung cancer				Gastric cancer			
	Univariate		Multivariate		Univariate		Multivariate	
	**Hazard ratio (95% CI)**	**p-value**	**Hazard ratio (95% CI)**	**P-value**	**Hazard ratio (95% CI)**	**P-value**	**Hazard ratio (95% CI)**	**p-value**
Age	1.05 (1.03–1.07)	<0.001	1.05 (1.03–1.07)	<0.001	1.06 (1.04–1.07)	<0.001	1.05 (1.03–1.07)	<0.001
Sex (Ref: Female)	1.66 (1.03–2.69)	0.038			2.30 (1.50–3.50)	<0.001	2.32 (1.51–3.56)	<0.001
Hypertension	2.58 (1.13–5.91)	0.025			2.28 (1.33–3.91)	0.003		
Renal disease					2.87 (1.26–6.51)	0.012		
Chronic pulmonary disease	2.12 (1.24–3.64)	0.006			3.69 (1.93–7.04)	<0.001	2.39 (1.24–4.61)	0.009
Mood disorder	3.87 (1.22–12.25)	0.021	3.45 (1.09–10.94)	0.036	4.20 (1.34–13.21)	0.014	4.46 (1.37–14.51)	0.013
Parkinson disease					4.09 (1.01–16.53)	0.048		
Cerebrovascular disease	3.36 (1.69–6.69)	0.001	2.18 (1.07–4.43)	0.032	2.91 (1.67–5.08)	<0.001		

CI: confidence interval
